# Elucidating the role of the gastrointestinal microbiota in racial and ethnic health disparities

**DOI:** 10.1186/s13059-020-02117-w

**Published:** 2020-08-03

**Authors:** Doratha A. Byrd, Tiffany L. Carson, Faustine Williams, Emily Vogtmann

**Affiliations:** 1 Division of Cancer Epidemiology and Genetics, National Cancer Institute, National Institutes of Health, Bethesda, MD USA; 2 Division of Preventive Medicine, Department of Medicine, School of Medicine, University of Alabama at Birmingham, Birmingham, AL USA; 3 O’Neal Comprehensive Cancer Center at UAB, University of Alabama at Birmingham, Birmingham, AL USA; 4 Division of Intramural Research, National Institute on Minority Health and Health Disparities, National Institutes of Health, Bethesda, MD USA

## Introduction

In the United States (US), racial and ethnic disparities in health outcomes persist despite advancement in biomedical technology, treatments, and public health interventions [1]. Historically, understanding the underlying causes of these disparities has been difficult. Racial and ethnic disparities are not attributable to a single factor, such as genetics, but to multi-factorial and complex factors known collectively as social determinants of health, which involve psychosocial, socioeconomic, cultural, dietary/lifestyle, healthcare-/policy-related, and other environmental factors [2, 3]. The term “race” was developed as a social construct rooted in justifications for oppression and discrimination on the basis of phenotypic features. Despite its roots, adverse events based on one’s race and their biological consequences are real, though exposures strongly correlated with racial identity are often difficult to measure and quantify with conventional research tools. The term “ethnicity” captures differences in nationality and in cultural and social groups, but groupings are usually broad and may not capture all details relevant to studying health disparities [2]. Despite limitations of the terms “race” and “ethnicity,” epidemiologic studies consistently demonstrate the persistence of racial/ethnic disparities even after adjustment for and stratification by biologically plausible, albeit often crudely measured, confounders and effect measure modifiers [4]. Clearly, there is an urgent need to understand the mechanisms whereby identifying as a certain race or ethnicity confers real biological consequences.

The gastrointestinal microbiota, herein focused on the trillions of bacteria residing in the oral cavity and gut, is presumably related to the etiology of multiple health conditions with racial/ethnic differences in incidence and survival. Many exposures that tend to differ across racial/ethnic groups may act individually and collectively to influence gastrointestinal microbial composition. For example, previous epidemiologic studies suggested racial/ethnic differences in some estimated dietary/lifestyle exposures (e.g., alcohol consumption) [5]. These exposures individually may explain some observed racial/ethnic differences in health outcomes, but it is clear that other unmeasured or poorly measured factors also play a role. Given that the oral and gut microbiota may reflect known and unknown race/ethnicity-related exposures, it is plausible that the gastrointestinal microbiota may be a useful target for addressing disparities. Herein, we discuss issues pertaining to studying racial/ethnic differences in the oral and gut microbiota and potential implications for addressing disparities.

## Genetics and the microbiota

Although it is well known that race and ethnicity are poor proxies for genetic ancestry, these factors may be somewhat correlated. Therefore, host genetics may marginally contribute to racial/ethnic differences in gastrointestinal microbial composition and functionality. Findings from genome-wide association studies of the microbiota have been inconsistent thus far. For example, a study conducted within 416 predominantly white British adult twin pairs from the TwinsUK cohort suggested that specific fecal microbial taxa may be influenced by host genetics [6]. In contrast, in a study of 1046 individuals with varied ancestral origins in Israel, there was little impact of host genetics on the fecal microbiota, whereas environmental factors more strongly contributed to fecal microbial composition [7]. These studies taken together, in addition to others not included here, suggest that it is likely that host genetics play a smaller role in shaping gastrointestinal microbiota than environmental exposures.

In order to understand the role of host genetics, if any, in associations between race and ethnicity and the gastrointestinal microbiota, it is essential to conduct genome-wide association studies with microbial data among diverse populations. In 2016, 81% of the existing genome-wide association study data were generated from individuals of European ancestry and only 3% from individuals of African ancestry and 0.05% from indigenous populations [8]. In order to have sufficient power to disentangle the contributions of host genetics and environmental exposures to oral and gut microbial composition, recruitment of large, diverse study populations comprising individuals from all ancestral groups must be a priority.

## Previous studies of racial and ethnic differences in the microbiota

The terms “race” and “ethnicity” likely encompass complex interactions of internal and external exposures that, in turn, affect gastrointestinal microbial composition. This is supported by accumulating evidence suggesting microbial differences by racial/ethnic group [9, 10]. In an investigation of associations of ethnic background with fecal microbial composition among 2084 individuals living in Amsterdam, Netherlands, comprising 439 Dutch, 367 Ghanaian, 280 Moroccan, 197 Turk, 443 African Surinamese, and 358 South-Asian Surinamese participants, after adjustment for potential confounders, ethnicity was strongly associated with alpha diversity, beta diversity, and the relative abundances of certain taxa (e.g., *Bacteroides*) [9]. In an investigation of racial differences in the oral microbiota in a subset of the Southern Community Cohort based in the Southeastern region of the US (*N* = 1058 African Americans, *N* = 558 European Americans), African Americans were more likely than European Americans to have higher alpha diversity, differences in overall microbial composition, and differences in the relative abundance of certain taxa (e.g., *Bacteroidetes*). In this study, African Americans were classified by both self-reported race and percentage of genetic African ancestry, and the microbial differences were consistent using both metrics [10].

While studies of racial/ethnic microbial differences have important implications, many of these studies had limitations that should be adequately addressed in future studies. Some of these limitations included small sample sizes, absence of adjustment/stratification for important confounders/effect measure modifiers, and no detailed investigations into other psychosocial, socioeconomic, genetic, environmental, and other exposures that may be associated with self-reported racial/ethnic classification. For instance, in the Southern Community Cohort study mentioned previously [10], oral health was crudely measured with the metric “numbers of teeth lost,” based on data with a high percentage of missing values [11]. Strong evidence suggests that, on average, African Americans have a higher prevalence of periodontal disease than other racial groups [12]. Periodontal disease may be attributable to complex and difficult-to-measure differences in socioeconomic and other social factors occurring over the life course that contribute to a lack of health care access and utilization. Therefore, oral health metrics and their associated oral microbiota may be proxies for these complex exposures. To contextualize findings from studies investigating racial differences in the oral microbiota, it is crucial to carefully measure and assess the role of oral health and related metrics as possible contributors to observed racial oral microbiota differences.

## Direction of future studies

A critical first step for future microbiota studies is to recruit highly diverse, representative participants, thus avoiding biases similar to those of the historically homogenous host genetics studies described above [8]. To do this, when designing microbiota studies, investigators should avoid convenience sampling methods for participant recruitment and instead conduct probability sampling targeted toward having adequate representation of all racial and ethnic groups, particularly typically underrepresented populations. This strategy helps to address some issues with volunteer self-selection bias (e.g., volunteers may have different social determinants of health characteristics from those who do not volunteer). For example, the annual National Health and Nutrition Examination Survey (NHANES) ascertains the health and nutritional status of children and adults via questionnaires and biospecimen collection in a nationally representative sample of approximately 5000 individuals [13]. Because of their oversampling of targeted groups of individuals, NHANES has become an invaluable resource for reliably estimating various US exposures, including those pertaining to social determinants of health. Thus, study populations like those represented in NHANES are particularly suitable for future collection of oral and fecal samples to characterize the gastrointestinal microbiota of a representative portion of the US population. But even on a smaller scale (e.g., clinic-based microbiota studies), it is essential that investigators focus on recruiting diverse individuals using strategies tailored as appropriate toward specific populations to ensure a representative study population.

There are immense gaps in knowledge pertaining to describing and understanding underlying causes of racial/ethnic differences in the gastrointestinal microbiota and the role of these differences in disparities. Filling existing gaps in knowledge requires the use of new, large, representative cross-sectional studies with fecal/oral samples in populations inclusive of individuals with diverse social determinants of health (e.g., NHANES). These studies should not only focus on racial/ethnic differences in the microbiota, but also emphasize the meticulous identification of exposures that may be driving observed racial/ethnic differences (e.g., diet, oral health, socioeconomic status). It is also urgent that we develop well-powered, deeply phenotyped, longitudinal cohorts among underrepresented populations with biosamples to be capable of prospectively assessing associations between the oral and fecal microbiota with disease risk. Currently, there is a field-wide lack of availability of gastrointestinal microbiota biospecimens (particularly of feces) collected with adequate time elapsed to follow individuals and observe their disease incidence (e.g., cancer). As expected, given the largely homogenous composition of most established cohorts in the US, these issues will likely adversely affect the future study of the microbiota among individuals from underrepresented populations in particular. In addition, birth cohorts with regular biospecimen collection should be developed that follow participants from early life to adulthood, assessing early- (e.g., delivery method, childhood trauma), mid-, and late-life exposures in detail to inform the interrelationships of these exposures with the microbiota and disparities. Notably, in longitudinal studies, it is important to use a multidisciplinary approach to improve study retention and follow-up among underrepresented populations so that diversity is maintained over the course of follow-up. Both cross-sectional and longitudinal studies should also emphasize interdisciplinary approaches to disentangling associations with related genetic, psychosocial, cultural, geographical, healthcare-/policy-related, diet/lifestyle, and environmental exposures. Collectively, implementation of these studies is critical to understanding the role of environmental factors in racial/ethnic microbiota differences and their collective roles in disparities.

If racial/ethnic differences in microbial composition and microbiota-disease associations persist after rigorous replication, we should develop thoughtful public health and clinical interventions targeted toward beneficially modifying the microbiota among high-risk populations. Though much research is still needed to understand what characterizes a “healthy” microbiome across diverse populations, investigators should start preparing now to advocate for the recruitment of diverse individuals into the ensuing intervention studies and randomized clinical trials. Investigators should engage in collaborative research partnerships involving community members and scientists from diverse backgrounds to (1) design and implement culturally sensitive/appropriate interventions and treatments and (2) develop clinical microbiota guidelines that not only incorporate phenotypic features, but also environmental, sociocultural, and physiological factors.

## Conclusions

In conclusion, as racial/ethnic disparities in health outcomes persist over time, there is urgency for race/ethnicity-focused microbiota research that captures interrelationships with exposures amenable to public health and clinical interventions. Future endeavors in these efforts will be particularly meaningful toward reducing disease burden among those populations that are disproportionately affected.
